# Anserine/Carnosine Supplementation Suppresses the Expression of the Inflammatory Chemokine CCL24 in Peripheral Blood Mononuclear Cells from Elderly People

**DOI:** 10.3390/nu9111199

**Published:** 2017-10-31

**Authors:** Yoshinori Katakura, Mamoru Totsuka, Etsuko Imabayashi, Hiroshi Matsuda, Tatsuhiro Hisatsune

**Affiliations:** 1 Faculty of Agriculture, Kyushu University, Higashi-ku, Fukuoka 812-8581, Japan; katakura@grt.kyushu-u.ac.jp; 2 Department of Applied Biochemistry, Graduate School of Agriculture and Life Sciences, The University of Tokyo, Tokyo 113-8657, Japan; atotuka@mail.ecc.u-tokyo.ac.jp; 3 Integrative Brain Imaging Center (IBIC), National Center of Neurology and Psychiatry, Tokyo 187-8551, Japan; embysh@ncnp.go.jp (E.I.); matsudah@ncnp.go.jp (H.M.); 4 Department of Integrated Biosciences, Graduate School of Frontier Sciences, The University of Tokyo, Kashiwa 277-8562, Japan

**Keywords:** Alzheimer’s disease, verbal memory, cognitive function, anserine and carnosine, RCT, elderly people, inflammatory chemokine, CCL24

## Abstract

Our goal was to determine whether anserine/carnosine supplementation (ACS) suppresses chemokine levels in elderly people. In a double-blind randomized controlled trial, volunteers were assigned to the ACS or placebo group (1:1). Sixty healthy elderly volunteers (active, *n* = 30; placebo, *n* = 30) completed the study. The ACS group was administered 1.0 g of anserine/carnosine (3:1) for 3 months. A microarray analysis and subsequent quantitative real-time polymerase chain reaction (qRT-PCR) analysis of peripheral blood mononuclear cells (PBMCs) showed decreased expression of CCL24, an inflammatory chemokine (*p* < 0.05). Verbal memory, assessed using the Wechsler memory scale–logical memory, was preserved in the ACS group. An age-restricted sub-analysis showed significant verbal memory preservation by ACS in participants who were in their 60s (active, *n* = 12; placebo, *n* = 9; *p* = 0.048) and 70s (active, *n* = 7; placebo, *n* = 11; *p* = 0.017). The suppression of CCL24 expression was greatest in people who were in their 70s (*p* < 0.01). There was a significant correlation between the preservation of verbal memory and suppression of CCL24 expression in the group that was in the 70s (Poisson correlation, *r* = 0.46, *p* < 0.05). These results suggest that ACS may preserve verbal episodic memory, probably owing to CCL24 suppression in the blood, especially in elderly participants.

## 1. Introduction

The number of people living with dementia worldwide was estimated to be 47 million in 2015, and Alzheimer’s disease (AD) is the most common form of dementia [1]. Diagnosis and intervention at early stages of dementia may greatly reduce the numbers of individuals who progress to developing AD. The onset of symptoms typically begins with a subtle decline in memory and progresses to global deterioration. Lifestyle improvements, including increased intellectual and physical activity, and nutritional improvement [2,3,4,5], that decelerate this process may delay or prevent the onset of AD. Among them, dietary improvements can help decelerate a decline in memory and may prevent AD [4]. One efficient solution is to use food supplements based on natural products which would have minimal side effects [5]. Here, we investigated the effects of dietary supplementation with anserine (beta-alanyl-3-methyl-l-histidine) and carnosine (beta-alanyl-l-histidine), referred to as imidazole-containing dipeptides, in healthy elderly people.

Carnosine is an endogenous dipeptide consisting of beta-alanine and histidine, which is present in the millimolar range in skeletal muscle and in the one hundred micromolar range in the vertebrate brain [6]. Carnosine is cleaved by carnosinase to beta-alanine and histidine. In rodents, serum carnosinase activity is low, so carnosine administration elevates plasma carnosine levels. However, in humans, high serum carnosinase activity limits the biomedical effectiveness of carnosine supplementation [6]. Anserine is a natural carnosine derivative that is not cleaved by carnosinase. Anserine (beta-alanyl-3-methyl-l-histidine) is a methylated form of carnosine that is present at high levels in the breast skeletal muscle of chicken. Owing to their identical chemical structures, with the exception of anserine methylation, anserine and carnosine have equivalent reported physiological functions [6]. At present, anserine alone is not commercially available, though we were able to obtain an anserine/carnosine mixture prepared from chicken meat. In clinical studies, Szcześniak et al. [7] and our group [8,9] have shown that anserine and carnosine supplementation (ACS) has beneficial effects on cognitive function in elderly people.

In this study, we elucidated the mechanism underlying the preservation of verbal episodic memory in elderly people, as evaluated by the Wechsler memory scale–logical memory (WMS-LM) [10]. Normal aging is associated with an increase in inflammatory chemokines, such as eotaxin 1 [11,12], in the blood, and this elevation is related to cognitive decline [11]. Therefore, to elucidate the effect of ACS, we examined the expression levels of chemokines in blood from participants using a microarray analysis followed by a quantitative real-time polymerase chain reaction (qRT-PCR) analysis, and assessed its role in the suppression of memory decline in elderly people.

## 2. Materials and Methods

### 2.1. Participants

Sixty-nine healthy volunteers (41–78 years of age) were enrolled in this study between June 2012 and November 2013 from the Tokyo metropolitan area, Japan [9]. All subjects gave their informed consent for inclusion before they participated in the study. The study was conducted in accordance with the Declaration of Helsinki, and the protocol was approved by the Ethics Committee of The University of Tokyo (#13-57). The participants were required to visit the study site twice, 3 months apart. Written informed consent was obtained from all participants. Participants with the following indications were excluded from this study: (1) individuals with a neuropsychiatric disorder or head injury; (2) individuals with a local lesion, such as a brain tumor or cerebral infarction, which could affect cognitive function; and (3) individuals with metal or electrical implants or claustrophobia that could prevent magnetic resonance image (MRI) scanning. The participants were randomized to the ACS or placebo group. RCT assignment to the ACS or placebo group was performed by Imepro Inc., (Tokyo, Japan), and was determined by age and sex. All clinical and coordinating personnel and participants were blinded to the group assignments for the duration of the study. The study was approved by the Ethics Committees of the University of Tokyo and of the National Center of Neurology and Psychiatry in 2012. Sixty participants ended this study.

### 2.2. Test Formulae

The test formula was a powder containing anserine and carnosine (3:1) derived from chicken meat, provided by NH Foods Ltd., Ibaraki, Japan. Participants in the ACS group received doses of the imidazole dipeptide formula (500 mg/dose) twice-daily. The safety of this formula was previously verified by two independent studies [13,14]. The placebo formula contained an equivalent amount of essential amino acids to those found in the test formula (43 mg/day l-lysine) and 150 mg/day l-histidine, because the enzymatic digestion of carnosine (250 mg/day) generates l-histidine (150 mg/day) and beta-alanine. Both treatments were granular solids taken orally over a 3-month period. All sixty participants reported to take more than 80% of test formulae during the test period. Absorption of imidazoledipeptide in elderly people has been confirmed in a separate test (data not shown).

### 2.3. Inventory of Food Intake during the 3-Month Test Period

A dietary survey was conducted using a semi-quantitative method, as reported elsewhere [15]. At the time of follow-up, the participants filled out a self-administered questionnaire on the frequency of animal meat (chicken, pork, and beef) and fish meat (red meat fish represented by tuna, white fish by salmon, blue-back fish by mackerel, and eel) intake over the previous 3 months. In Japan, this 3-item fish consumption inquiry is popular, and salmon is classified as a white fish even though it is pink in color. The representative fish for each category was based on the national consumption survey. The average anserine and carnosine concentrations in these meats and fish were obtained from Boldyrev et al. [6], and dietary intake was estimated from the responses to the questionnaire.

### 2.4. Cognitive Testing

The following cognitive evaluation tools and self-report questionnaires were used to assess the effects of ACS on cognitive function, mental status, and general health: (1) the Japanese version of the Wechsler memory scale–revised logical memory immediate recall (WMS-LM1) and delayed recall (WMS-LM2) tests [10]; and (2) the Japanese version of a cognitive subscale of the Alzheimer’s disease assessment scale (ADAScog) [16,17]. Mood and subjective states were assessed by the Japanese version of the Beck depression inventory (BDI) [18,19]. In addition, mental and physical functional well-being was assessed by the medical outcomes study, 36-item short form (SF-36) [20,21]. The mental health component summary (MCS) score and physical health component summary (PCS) score were calculated, with higher scores indicating better functioning. The cognitive and psychological tests were performed under double-blind conditions. A mini mental state examination (MMSE) was also conducted to assess baseline cognitive function [22].

### 2.5. Microarray Analysis

Peripheral blood mononuclear cells (PBMCs) from volunteers were used for total RNA extraction using a PAXgene Blood RNA Kit (Qiagen, Valencia, CA, USA). The total RNA quality was assessed using the Agilent system according to the manufacturer’s protocol. We eliminated several RNA samples from the two test groups owing to poor quality. cRNA was labeled using low input quick amp labeling (Agilent Technologies, Santa Clara, CA, USA), and hybridized onto a human whole genome oligo DNA Microarray Ver2.0 (Agilent Technologies, Santa Clara, CA, USA), as described elsewhere [23]. Relative hybridization intensities and background hybridization values were calculated using feature extraction Software (Agilent Technologies, Santa Clara, CA, USA). The raw signal intensities of 120 samples were normalized by the quantile algorithm in the “preprocessCore” library package [24] in the Bioconductor software [25]. The log fold-change (=Delta) of each probe in paired samples was calculated, and a Student’s *t*-test was applied using the MeV software [26]. We obtained 2650 genes that were significantly differentially expressed (*p* < 0.05) using a Student’s *t*-test between the Delta of placebo and active samples (exposed to ACS). One hundred and fifty-one significant genes were implicated in the “immune response” or “inflammatory response” in the gene ontology annotation.

### 2.6. qRT-PCR

cDNA was prepared using ReverTra Ace (Toyobo, Osaka, Japan). qRT-PCR was performed using the KAPA SYBR Fast qPCR Kit (KAPA Biosystems, Woburn, MA, USA) and Thermal Cycler Dice Real Time System TP-800 (Takara, Shiga, Japan). Samples were analyzed in triplicate, and gene expression levels were normalized to the corresponding β-actin level. The PCR primers for CCL24 were as follows: Forward primer: 5′-CCAGCCTTCTGTTCCTTGGTG-3′; reverse primer: 5′-AACTGCTGGCCCTTCTTGGT-3′.

## 3. Results

### 3.1. Participants

We used the data from all participants who completed the study. The participants were randomly assigned to the ACS and placebo groups. Sixty participants completed both the baseline and follow-up tests. The group characteristics are summarized in Table 1. The two groups did not differ significantly with respect to age, sex, body-mass index (BMI), education, or MMSE score, although there was a slight age difference between the two groups owing to random drop-out of participants. The baseline MMSE score of all the participants was greater than 24.

The purpose of this study was to test the effect of anserine/carnosine supplementation, but these dipeptides are also obtained from the diet. To estimate the daily intake from the diet, we estimated the anserine + carnosine intake from animal and fish meats using a 7-item meat intake frequency questionnaire (Table 2). There was no statistical difference in the daily anserine/carnosine intake from the diet between the two groups. Taking the dietary intake into account, the ACS group took in approximately 3 times more anserine/carnosine than the placebo group.

### 3.2. Microarray and qRT-PCR Analysis

**Figure 1:**
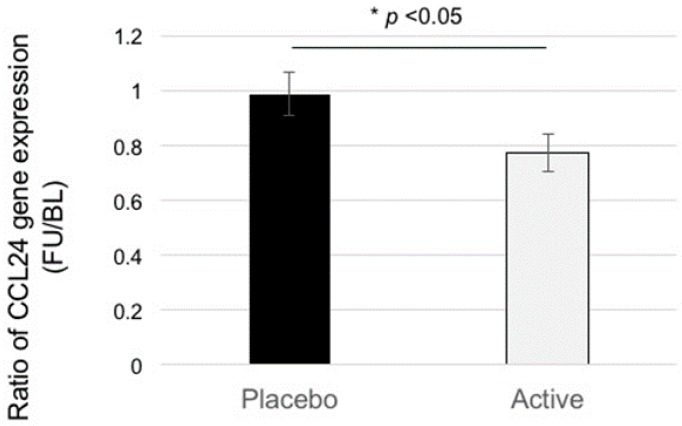
Anserine/carnosine supplementation (ACS) suppresses CCL24 expression in peripheral blood mononuclear cells (PBMCs) (all participants). Placebo, active. Data = Ave ± SEM. * Student’s two-tailed *t*-test, *p* < 0.05. BL, baseline; FU, follow-up.

We performed a microarray analysis of the blood samples obtained from participants at the baseline and follow-up timepoints. After normalization of the gene expression between the samples, we calculated the log2 fold-change (Delta) between the pre-intake and post-intake blood samples as altered expression values. A student’s *t*-test was conducted between the Deltas of the ACS and placebo groups, which showed that 2650 genes were significantly differentially expressed (*p* < 0.05). Of these, we focused on the genes (151) categorized as “inflammatory-related genes including soluble factors”, such as chemokines, which would be expected to affect neural tissues and/or cerebrovascular cells, and further selected six genes (Chemokine (C-C motif) ligand 24 (CCL24), platelet-derived growth factor subunit B (PDGFB), interleukin (IL)-17B, IL-28A, IL-4, and IL-6) with a relatively higher expression level (signal intensity ≥ 100). Of these, we selected the inflammatory chemokine CCL24 for further analysis because CCL24 showed the highest level of expression. Figure 1 shows the changes in CCL24 expression in PBMCs. We found a significant difference between the two test groups (*p* < 0.05).

### 3.3. Cognitive Tests

To evaluate cognitive function at baseline and follow-up, we performed two neuropsychological tests (Table 3). For the WMS-LM2 test, used to assess the delayed recall of verbal memory, we used two different stories (story A and story B) for the baseline and follow-up tests, respectively, to prevent possible memorization of the story. Data were analyzed using a two-way repeated measures ANOVA (Time (baseline or follow-up) × Variant (ACS or placebo)). The Time × Variant interaction was *p* = 0.078. In the WMS-LM1 test, used to assess the immediate recall of verbal memory, we did not see any difference between the two groups (*p* = 0.31). In the ADAScog test, the Time × Variant interaction was not significant (*p* = 0.47). In the PCS score from the SF-36, the Time × Variant interaction showed a trend towards significance (*p* = 0.08). In the MCS score from the SF-36, the Time × Variant interaction was not significant (*p* = 0.30). In the BDI test, the Time × Variant interaction showed a weak trend towards significance (*p* = 0.14).

### 3.4. Age-Restricted Sub-Analysis (Participants in Their 60s and 70s)

**Figure 2:**
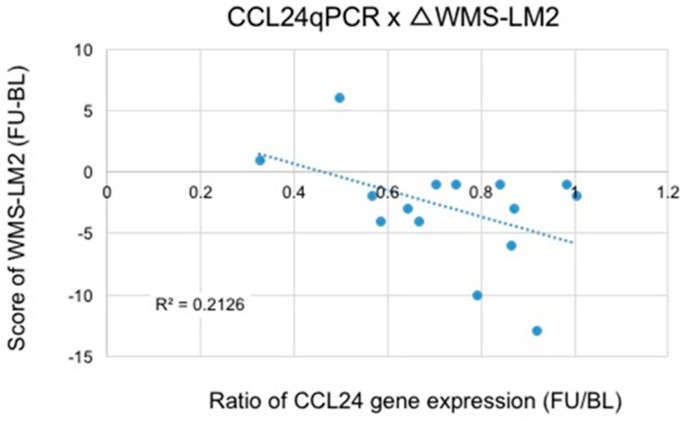
Correlation between the CCL24 suppression and the improvement of the score of the Wechsler memory scale–logical memory with delayed recall of verbal episodic memory (WMS-LM2) (in participants in their 70s. Poisson correlation analysis (*R*^2^ = 0.213, *r* = 0.46, *p* < 0.05). BL, baseline; FU, follow-up.

**Figure 3:**
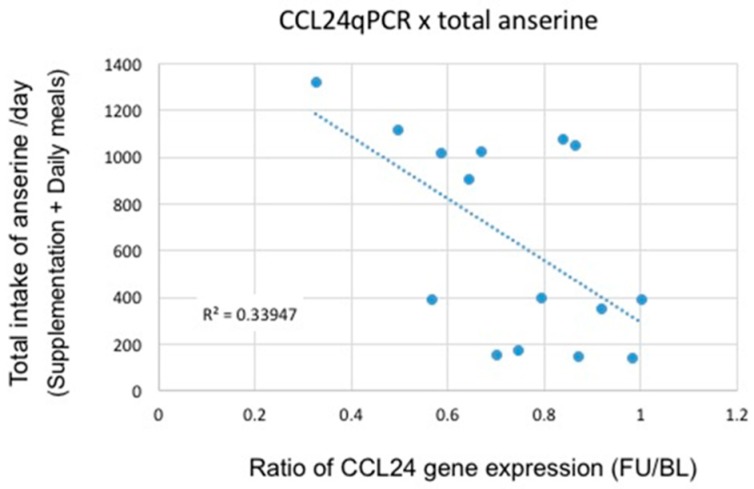
Correlation between the CCL24 suppression and total anserine intake/day in participants in their 70s. Poisson correlation analysis (*R*^2^ = 0.3395, *r* = 0.58, *p* < 0.025). BL, baseline; FU, follow-up.

**Figure 4:**
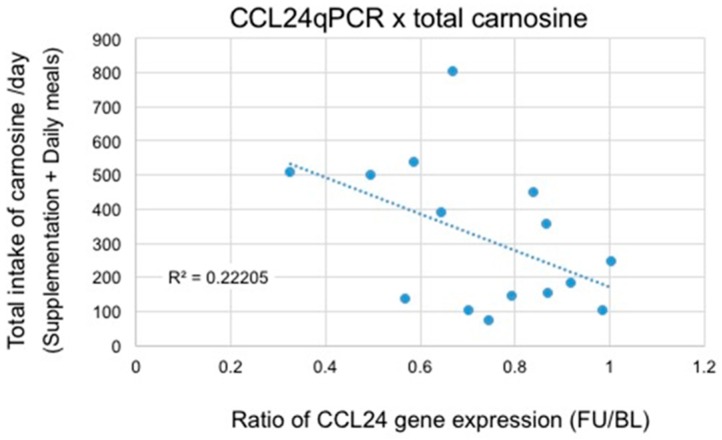
Correlation between CCL24 suppression and total carnosine intake/day in participants in their 70s. Poisson correlation analysis (*R*^2^ = 0.222, *r* = 0.47, *p* < 0.05). BL, baseline; FU, follow-up.

We conducted age-restricted sub-analyses examining (1) participants in their 60s (Table 4); and (2) participants in their 70s (Table 5). Table 4 shows the results from psychological test scores and CCL24 expression levels in the participants in their 60s. With regard to the WMS-LM2 test, we found a significant difference between the two groups using a two-way repeated measures ANOVA (Time (baseline or follow-up) × Variant (ACS or placebo), *p* = 0.048). We also observed a trend towards significance in the suppression of CCL24 expression (*p* = 0.14). For participants in their 70s (Table 5), we found a significant difference in both the WMS-LM2 test (*p* = 0.017) and CCL24 expression (*p* = 0.006) between the two groups. There was a significant correlation between the WMS-LM2 test score and the ratio of CCL24 expression (Figure 2; *R* = 0.45, *p* < 0.05). Using data from daily anserine/carnosine intake from participants, we also plotted total daily intake of the two dipeptides (anserine and carnosine) versus the ratio of CCL24 expression in participants who were in their 70s (Figure 3 and Figure 4). We found a strong correlation between the total daily anserine intake and the ratio of CCL24 expression, and a significant correlation between the total daily carnosine intake and CCL24 expression in participants who were in their 70s.

## 4. Discussion

In this study, we found that ACS significantly suppressed the expression of a blood inflammatory chemokine, CCL24, in elderly participants (Figure 1). In the older participants, i.e., participants who were in their 70s, the suppression of CCL24 expression was positively correlated with the preservation of verbal episodic memory (Figure 2). Although anserine and carnosine are also taken in in the daily diet, the suppression of CCL24 expression correlated well with the daily total intake (total of supplementation + daily meal) of anserine and carnosine (Figure 3 and Figure 4). Prior to selecting this inflammatory chemokine, CCL24, for further examination, we performed a genome-wide microarray analysis using the human whole genome oligo DNA Microarray Ver2.0 (Agilent Technologies). We detected a significant difference between the ACS and placebo groups in 2650 genes. Among the genes that showed significant differences, we focused on the 151 genes categorized as “inflammatory related genes including soluble factors”, such as chemokines, which would be expected to affect memory decline in elderly people. Then, we focused on six genes (CCL24, PDGFB, IL-17B, IL-28A, IL-4 and IL-6) that had a relatively higher expression level (signal intensity ≥ 100). Finally, we selected CCL24 because it showed the highest level of expression.

CCL24 is a member of the eotaxin family (eotaxin-2) [12]. Another eotaxin, CCL11 (eotaxin-1), is known to be involved in neurodegeneration [11]. Increased levels of CCL11 are associated with a decline in neurogenesis and impaired cognition and memory in mice [11]. Furthermore, elevated levels of CCL11 have been observed in several neurodegenerative diseases in humans, such as Alzheimer’s disease and Huntington’s disease [12]. Thus, CCL24 as well as CCL11 might be involved in aging-associated neurodegenerative diseases. In elderly individuals, it is possible that the intake of anserine and carnosine is beneficial and can preserve cognitive function, such as memory, by suppressing blood inflammatory chemokines that may damage neurovascular as well as neural function.

Given that the delayed-recall tests of verbal memory, such as the WMS-LM2 test [27] or the free and cue selective reminding test (FCSRT) [28,29,30], are considered a sensitive battery for detecting cognitive decline in elderly people, our findings indicate that ACS may inhibit memory decline, as detected by the verbal memory test [8,9]. In line with our findings, Szcześniak et al. [7] have also suggested that ACS inhibits the decline in delayed recall, though not immediate recall, memory in elderly people, as assessed using the STMS [28]. In addition to the delayed-recall tests of verbal memory, we also observed a weak trend towards improvement in the WMS-1 for assessing the immediate recall of verbal memory (Table 5), and the BDI test for assessing the level of depression (Table 5) following ACS. However, we did not detect a significant correlation between these improvements and the suppression of CCL24 expression. The delayed-recall tests of verbal memory, which is dependent on hippocampal function, in elderly individuals may depend on the suppression of blood inflammatory reactions.

In this study, we detected significant decreases in CCL24 expression in the ACS group after supplementation with ACS, which agrees with our previous finding that ACS treatment decreases the production of IL-8 in elderly people [9], and carnosine treatment decreases the production of IL-8 in tumor necrosis factor (TNF)-treated cells [31]. How ACS suppresses the inflammatory reaction, neurovascular damage, and maintains brain blood flow is still unknown; thus, further studies are needed. In mammals, the concentration of imidazoledipeptide, carnosine or anserine, is in the high millimolar range in muscle tissues [6,32,33]. A beneficial effect of imidazoledipeptide on the cardiovascular system, including the smooth muscle cells of blood vessels, has been confirmed in various experimental animal models [34,35]. In our microarray-qRT-PCR assay, we detected a change in CCL24 expression in PBMCs. Recently, Zlokovic and colleagues elegantly demonstrated the role of brain blood vessel pericytes in enabling brain microcapillaries to maintain brain blood flow, and suggested that pericyte degeneration contributes to the etiology of Alzheimer’s disease [36,37]. It can be speculated that ingested anserine and carnosine suppress inflammatory reactions in the blood, and the damage to brain microcapillaries induced by pericytes; however, further studies are required to determine the precise mechanisms by which ACS affects brain function.

Our study has various limitations. The sample size was limited, and represented an age-based subgroup analysis from a larger original group that included data from younger participants. Age is an important risk factor in Alzheimer’s disease, in order to systematically defining age sensitivity/dependences we are in underway to plan next RCT study with a larger number of participants. In this study, we used a mixture of anserine and carnosine, and we did not establish which molecule was the most effective. While we tried to estimate the participants’ dietary intake of anserine and carnosine using a questionnaire, a better method is needed to estimate their levels more accurately.

## Figures and Tables

**Table 1:** Subject characteristics ^1^.

Demographics	Active Group	Placebo Group	*p* Value
Age	60.4 (2.1)	65.3 (1.6)	0.07
Sex (M/F)	10/20	10/20	-
BMI	21.0 (0.51)	21.1(0.86)	0.97
Years of education	14.9 (0.4)	14.9 (0.4)	0.995
MMSE	28.7 (0.3)	29.1 (0.2)	0.29
*APOE4*+/*APOE4−*	4/26	4/26	-

^1^ Data (average (Ave) with standard error of the mean (SEM)) are shown for 60 participants who completed the baseline and follow-up tests. BMI: body-mass index; MMSE: mini mental state examination.

**Table 2:** Estimated amount of anserine/carnosine from daily meals.

Imidazoledipeptide	Active Group	Placebo Group	*p* Value
Anserine	384 ± 45 ^1^	358 ± 48 ^1^	0.699
Carnosine	259 ± 25 ^1^	199 ± 19 ^1^	0.058

^1^ Data are represented by Ave ± SEM (mg)/day.

**Table 3:** Psychological test scores for all participants ^1^.

Test	Baseline		Follow-Up		Change		
	Active	Placebo	Active	Placebo	Active	Placebo	*p* Value ^2^
WMS-1 ^3^	14.8 (0.8)	14.4 (0.7)	12.2 (0.7)	12.1 (0.8)	−2.6 (0.7)	−2.4 (0.5)	0.39
WMS-2 ^3^	13.6 (0.8)	13.9 (0.6)	12.0 (0.7)	11.0 (0.8)	1.4 (3.0)	0.7 (3.0)	0.078 ^##^
ADAS	8.3 (0.7)	7.2 (0.8)	7.3 (0.8)	7.2 (0.9)	−0.9 (0.6)	−0.04 (0.8)	0.18 ^#^
SF-36 (PCS)	47.3 (1.5)	47.6 (1.2)	48.5 (1.4)	48.3 (1.5)	1.2 (1.3)	0.7 (1.4)	0.40
SF-36 (MCS)	50.4 (1.8)	49.5 (1.4)	52.2 (1.4)	51.5 (1.2)	1.7 (1.2)	2.0 (1.1)	0.44
BDI	9.8 (1.2)	9.5 (1.2)	6.9 (0.9)	7.7 (0.9)	−2.9 (1.0)	−1.8 (0.9)	0.19 ^#^

^1^ Data are represented by Ave (SEM). Significant deterioration in the placebo group at the follow-up test (*p* < 0.01, by Student’s two-tailed *t*-test); ^2^ To evaluate the effect of ACS on the protection against cognitive decline, data from the two groups (active and placebo) were analyzed by a one-tailed *t*-test; ^##^ shows a trend towards significance (*p* < 0.1), ^#^ shows a weak trend towards significance (*p* < 0.2); ^3^ We utilized story A in the baseline test and story B in the follow-up test. In the Japanese version of Wechsler memory scale (WMS) [10], story B is relatively difficult than story A, which may cause the difference of the test score between baseline and follow-up tests. ADAS: Alzheimer’s disease assessment scale; PCS: physical health component summary; MCS: mental health component summary. BDI: Beck Depression Inventory.

**Table 4:** Psychological test scores and CCL24 expression for participants in their 60s ^1^.

60s Group	Baseline		Follow-Up		Change		
	Active	Placebo	Active	Placebo	Active	Placebo	*p* Value ^2^
WMS-1 ^3^	13.5 (0.8)	13.4 (0.8)	11.3 (0.6)	10.2 (0.7)	−2.3 (0.9)	−3.2 (0.6)	0.31
WMS-2 ^3^	12.1 (0.8)	12.7 (0.6)	11.2 (0.6)	9.2 (0.6)	−0.9 (0.7)	−3.4 (0.5)	0.048 *
ADAS	9.5 (0.8)	7.6 (0.7)	8.1 (0.7)	6.4 (0.7)	−1.4 (0.6)	−1.2 (0.9)	0.47
SF-36 (PCS)	48.6 (1.3)	49.6 (1.0)	52.1 (0.4)	48.0 (2.1)	3.4 (1.4)	−1.6 (1.5)	0.08 ^##^
SF-36 (MCS)	53.3 (1.6)	51.0 (1.6)	52.1 (1.4)	51.6 (1.0)	−1.1 (1.2)	0.6 (1.6)	0.30
BDI	9.4 (1.2)	8.9 (1.1)	6.6 (0.9)	8.6 (0.7)	−2.8 (0.8)	−0.3 (1.1)	0.14 ^#^
CCL24 post/pre	-	-	-	-	0.86 (0.07)	1.09 (0.09)	0.14 ^#^

^1^ Data are represented by Ave (SEM). Significant deterioration in the placebo group at the follow-up test (*p* < 0.05, by Student’s two-tailed *t*-test); ^2^ To evaluate the effect of ACS on the protection of cognitive decline, data from the two groups (active and placebo) were analyzed by a one-tailed *t*-test; * *p* < 0.05, ^##^ shows a trend towards significance (*p* < 0.1), ^#^ shows a weak trend towards significance (*p* < 0.2); ^3^ We utilized story A in the baseline test and story B in the follow-up test. Story B is relatively more difficult than story A.

**Table 5:** Psychological test scores for participants in their 70s ^1^.

70s Group	Baseline		Follow-Up		Change		
	Active	Placebo	Active	Placebo	Active	Placebo	*p* Value ^2^
WMS-1 ^3^	13.2 (0.7)	13.0 (0.7)	11.6 (0.8)	10.2 (0.7)	−1.6 (0.4)	−2.8 (0.5)	0.15 ^#^
WMS-2 ^3^	11.6 (0.7)	13.5 (0.8)	11.7 (0.9)	8.9 (0.9)	0.14 (3.0)	−4.55 (3.0)	0.017 *
ADAS	9.9 (0.6)	10.3 (0.7)	10.0 (1.1)	11.5 (0.7)	0.1 (0.6)	1.3 (0.9)	0.30
SF-36 (PCS)	45.4 (1.7)	46.7 (1.6)	42.3 (0.9)	46.7 (1.3)	−3.0 (1.4)	0.1 (1.5)	0.21
SF-36 (MCS)	55.3 (1.6)	50.3 (1.0)	57.2 (1.3)	53.7 (1.2)	1.9 (1.1)	3.3 (0.8)	0.28
BDI	11.6 (1.1)	8.7 (1.4)	7.1 (0.8)	6.5 (0.8)	−4.4 (0.8)	−2.3 (0.9)	0.18 ^#^
CCL24 post/pre	-	-	-	-	0.59 (0.03)	0.83 (0.03)	0.006 **

^1^ Data are represented by Ave (SEM). 74.1 ± 0.5 (*n* = 7) in the active group, 74.5 ± 0.4 (*n* = 11) in the placebo group; ^2^ To evaluate the effect of ACS on the protection of cognitive decline, data from the two groups (Active and Placebo) were analyzed by a one-tailed *t*-test; ** *p* < 0.01, * *p* < 0.05, ^#^ shows a weak trend towards significance (*p* < 0.2); ^3^ We utilized story A in the baseline test and story B in the follow-up test. Story B is relatively more difficult than story A.
